# Physical Examination Practices of Internal Medicine Residents: A Pilot Study

**DOI:** 10.7759/cureus.110721

**Published:** 2026-06-12

**Authors:** Jonathan Zaid, Jason Ojeda, Elizabeth Boyle, Christopher Henry, Rhea Powell, Maclain Capron, Michael Stillman

**Affiliations:** 1 Internal Medicine, Thomas Jefferson University, Philadelphia, USA; 2 Gastroenterology and Hepatology, Thomas Jefferson University Hospital, Philadelphia, USA; 3 College of Rehabilitation Sciences, Thomas Jefferson University, Philadelphia, USA

**Keywords:** bedside teaching, direct observation, graduate medical education, medical education, outpatient primary care

## Abstract

Background

Several foundational studies have demonstrated that Internal Medicine (IM) residency programs undervalue physical examination skills and infrequently provide direct clinical oversight of residents as they perform physical examinations. This lack of prioritization is consequential. While the literature assessing the accuracy and completeness of IM residents’ physical examinations is relatively narrow, certain studies have demonstrated worrisome deficiencies in their clinical skills.

Methodology

In this single-institution, exploratory, survey-based pilot study, we queried how IM residents perform examinations and their opinions of their own abilities and the adequacy of their training.

Results

Our most critical findings were that nearly all residents (98.3%) examined patients while they were fully clothed and 42.1% believed that such examinations were “adequate and accurate.” Only around half (51.7%) of respondents reported that supervising physicians routinely return to the examination room with them, and nearly 90% said they are rarely provided beside instruction. While a large majority felt that physical examinations help them develop differential diagnoses and diagnose their patients’ conditions, only 58.2% felt confident in their examination skills.

Conclusions

We hope that this preliminary work will lead to collaborations with other training programs in an effort to better understand the state of IM residents’ clinical training.

## Introduction

Internal Medicine (IM) residency programs have long under-emphasized the physical examination. Angus et al. asked 282 IM program directors (PDs) which two skills interns should possess [1]. Only 100 chose physical examination. When Stillman et al. queried IM residents about oversight by attending physicians [2], 33% had never been observed performing a physical examination, and 45% had been observed only once.

This lack of prioritization is consequential. Medical interns assessed by Ramani et al. failed to palpate abdomens, detect shifting dullness, distinguish between upper and lower motor neuron signs, and identify common murmurs [3]. Wray et al. found that IM residents’ admission examinations were commonly incorrect or incomplete [4]. Mangione et al., after demonstrating house officers’ inability to correctly identify common cardiac pathologies during auscultation, warned of a “downward spiral” in clinical skills [5].

While IM residents’ examination skills have been queried, their opinions of their own abilities and the adequacy of their training have not. In this exploratory pilot study, we surveyed residents in a single academic IM residency program to better understand the state of IM residents’ clinical training.

## Materials and methods

Four authors (MS, JZ, EB, and JO) developed a survey assessing residents’ examination styles, confidence in their examination skills, perceptions of the importance of physical examination, and satisfaction with their examination instruction. The instrument (Appendices), pertaining only to participants’ continuity clinics, was reviewed and edited by six former residents, followed by two authors (RP, CH) with expertise in qualitative research and assessment. Face and content validity were part of the initial review. Given that this was a novel survey that asked participants questions never addressed by prior authors, construct validity was difficult to assess.

The final survey was exempted from IRB review, loaded onto the SurveyMonkey platform, and then distributed to non-preliminary residents via three monthly emails between September and December 2025. Responses were gathered through March 2026, and then the survey was closed. Descriptive analysis was performed on the survey data, with counts and percentages being pulled directly from the survey platform.

Data analysis was conducted using SPSS version 30.0 (IBM Corp., Armonk, NY, USA). Participants were asked to report their year of residency, which for the purpose of analysis was grouped into PGY1 and PGY2/3. Likert scale data collected to assess confidence in and perceptions of physical examination utilized the five-point agreement Likert Scale. In the items assessing comfort, 1 indicated “Strongly Disagree,” 2 indicated “Somewhat Disagree,” 3 indicated “Neither Agree Nor Disagree,” 4 indicated “Somewhat Agree,” and 5 indicated “Strongly Agree.” For the purposes of analysis, the lower and upper values were grouped such that “Strongly Disagree” and “Somewhat Disagree” were combined into a “Disagree” category, “Strongly Agree” and “Somewhat Agree” were combined into an “Agree” category, and the central category of “Neither Agree Nor Disagree” was not grouped. Descriptive analysis was used to assess confidence. The associations between year of residency and other key variables were evaluated using chi-square tests for association. Notably, not every participant completed the survey in its entirety. Hence, we analyzed each question based on its number of responses and have provided those individual n values in both the text and the tables.

## Results

Overall, 70 residents responded to the survey (response rate = 63.1%; 70/111). Of the respondents, 50% were male, there was a near-even split between participants’ level of training (30% PGY1 (21/70), 34.3% PGY2 (24/70), 35.7% PGY3 (25/70)), 86.4% (57/66) were in the categorical track (13.6% (9/66) in primary care (PC)), and 66.2% (43/65) planned to pursue fellowships (13.9% (9/65) hospitalist, 9.2% (6/65) PC, and 10.8% (7/62) uncertain).

Nearly every participant (98.3%; 58/59) examined patients while they were fully clothed, with 86.4% (51/59) reporting that there was insufficient time for patients to gown. However, 32.2% (19/59) had been taught, and 40.1% (24/59) believed that examinations performed on clothed patients were “accurate and adequate.” Only 50% (30/60) reported that supervising physicians routinely returned to examination rooms with them, and 81.4% (48/59) said that they were “rarely” observed performing examinations or provided beside instruction (89.5%; 51/57).

**Figure 1:**
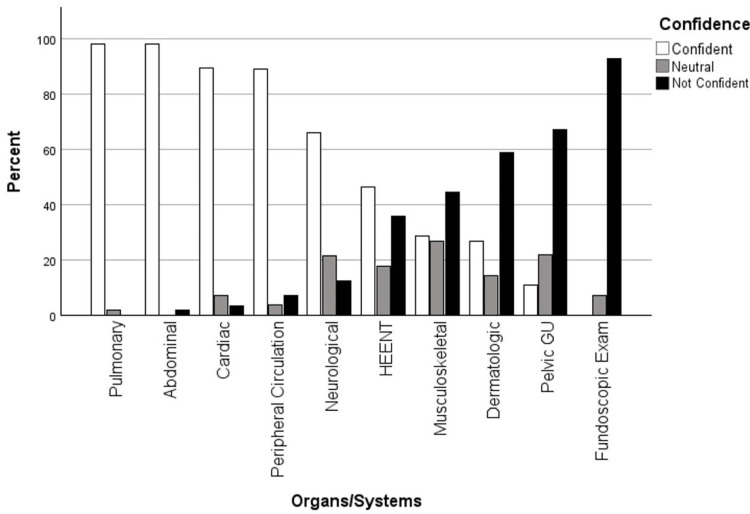
Organs/Systems confidence cluster bar graph. Strongly Agree and Agree were grouped into Confident, and Strongly Disagree and Disagree were grouped into Not Confident. Neither agree nor disagree was recoded into Neutral in this figure.

Large majorities agreed that physical examination helps them develop differential diagnoses (82.5%; 47/57) and diagnose their patients (78.9%; 45/57), though only 59% (33/56) felt confident in their examination skills. There was wide variability in respondents’ confidence in examining different organ systems (Figure 1), with nearly all participants feeling comfortable performing pulmonary and abdominal examinations (98.2%; 55/56 for both) and only small minorities feeling comfortable performing gynecologic (10.9%; 6/55), dermatologic (26.8%; 15/56), and musculoskeletal (28.5%; 16/56) examinations. A substantial majority felt their examination instruction was inadequate (64.3%; 36/56) and 92.9% (52/56) desired more, yet 30.3% (17/56) feared that seeking additional instruction may reflect poorly on them.

Survey questions were analyzed by level of training; responses offered by PGY1 residents were compared to the pooled responses from PGY2 and PGY3 residents. First-year trainees were no more likely than more senior residents to have attending physicians return to examination rooms with them, to be directly observed performing examinations, or to be given bedside instruction (Table 1).

**Table 1:** Residents’ direct observation and bedside instruction by level of training.

Question	Groups	Answer n (% of group)			Pearson’s chi-square, p-value
		Rarely	Half the Time	Routinely	
Physician observes my physical examination	PGY1 (n = 17)	14 (82.4)	3 (17.6)	0 (0.0)	χ^2^ = 0.415, p = 0.813
	PGY2 & 3 (n = 42)	34 (81.0)	7 (16.7)	1 (2.4)	
Physician gives bedside instruction	PGY1 (n = 16)	15 (93.8)	1 (6.3)	0 (0.0)	χ^2^ = 0.432, p = 0.511
	PGY2 & 3 (n = 41)	36 (87.8)	5 (12.2)	0 (0.0)	
Physician returns to the room with me	PGY1 (n = 17)	1 (5.9)	7 (41.2)	9 (52.9)	χ^2^ = 3.393, p = 0.183
	PGY2 & 3 (n = 43)	11 (25.6)	11 (25.6)	21 (48.8)	

Further, PGY1 respondents were no less likely than were the PGY2 and PGY3 participants to feel confident in their physical examination skills (62.5%; 10/16 vs. 57.5%; 23/40) or to believe that physical examination findings help refine differential diagnoses (87.5%; 14/16 vs. 80.5%; 33/41) and diagnose patients (81.3%; 13/16 vs. 78%; 32/41) (Table 2).

**Table 2:** Residents’ confidence in and reflections on physical examination by level of training.

Question	Groups	Answer n (% of group)			Pearson’s chi-square, p-value
		Agree	Neither Agree nor Disagree	Disagree	
Feel confident in physical examination skills	PGY1 (n = 16)	10 (62.5)	4 (25.0)	2 (12.5)	χ^2^ = 0.764, p = 0.683
	PGY2 & 3 (n = 40)	23 (57.5)	8 (20.0)	9 (22.5)	
My physical examination findings help refine a differential diagnosis	PGY1 (n = 16)	14 (87.5)	0 (0.0)	2 (12.5)	χ^2^ = 2.372, p = 0.305
	PGY2 & 3 (n = 41)	33 (80.5)	5 (12.2)	3 (7.3)	
My physical examination findings help diagnose my patients’ conditions	PGY1 (n = 16)	13 (81.3)	1 (6.3)	2 (12.5)	χ^2^ = 1.026, p = 0.599
	PGY2 & 3 (n = 41)	32 (78.0)	6 (14.6)	3 (7.3)	

Finally, our respondents, regardless of level of training, were equally unlikely to believe they are “provided adequate physical examination instruction” (37.5%; 6/16 for PGY1s; 35%; 14/40 for PGY2s and 3s), and nearly all desired additional instruction (100%; 16/16 for PGY1s; 90%; 36/40 for PGYs and 3s) (Table 3).

**Table 3:** Residents’ interest in additional physical examination instruction by level of training.

Question	Groups	Answer n (% of group)		Pearson’s chi-square, p-value
		True	False	
I am provided adequate physical examination instruction	PGY1 (n = 16)	6 (37.5)	10 (62.5)	χ^2^ = 0.031, p = 0.860
	PGY2 & 3 (n = 40)	14 (35.0)	26 (65.0)	
Enhanced physical examination instruction would help me develop	PGY1	16 (100.0)	0 (0.0)	χ^2^ =1.723, p = 0.189
	PGY2 & 3	36 (90.0)	4 (10.0)	

Participants detailed how feedback about their examination skills could be improved and which factors limit bedside instruction (Table 4). Large majorities of respondents reported that more real-time observation (80.7%; 46/57), more allotted time for each patient visit (79%; 45/57), and increased engagement by supervising physicians (64.9%; 37/57) would enhance the quality of their feedback. Nearly all residents (96.5%; 55/57) selected “lack of time for bedside teaching” as an impediment to bedside physical examination instruction.

**Table 4:** Residents’ perceptions of factors that would improve feedback about and limit instruction in physical examination. N = 57 participants answered these questions.

Question	Answers	n (%) of respondents
The following would improve and enhance the quality of feedback	More specific feedback	8 (14.0)
	More real time observation with feedback	46 (80.7)
	Increased use of standardized patients	8 (14.0)
	Increased engagement by supervising physicians	37 (64.9)
	More time allotted for each clinic visit	45 (79.0)
	Other	3 (5.3)
	None. The feedback I receive is sufficient	0 (0.0)
Which factors most limit bedside physical examination instruction	Lack of interest by residents	12 (21.1)
	Lack of interest by supervising physicians	12 (21.1)
	Lack of time for bedside teaching	55 (96.5)
	Lack of knowledge of physical examination skills by supervising physicians	7 (12.3)
	Other	2 (3.5)
	None. The instruction is sufficient	1 (1.8)

## Discussion

This single-institution pilot study is admittedly limited. However, our inquiry begins to interrogate IM residents’ attitudes toward physical examination, to document specific gaps in their examination skills, and to provide insights into current shortcomings in graduate-level outpatient medical education and how IM residency programs can improve physical examination instruction.

Our first finding is that respondents unequivocally desired improved examination instruction. It has long been known that there is a lack of bedside teaching in outpatient resident clinics. In Glen et al.’s study of observed interactions between Family Medicine (FM) attending physicians and residents [6], supervising physicians were brought to the bedside during only 32% of patient visits. In Williamson et al.’s analysis of teaching practices in an outpatient FM resident clinic [7], only 20% of the intern’s patients were seen by attending physicians, and that percentage dropped for patients being evaluated by second (12%) and third-year (13%) residents. In Brook et al.’s study of 15 IM university-affiliated practices [8], attending physicians examined only 2% of residents’ patients and explained treatments to those patients during only 11% of visits.

Although residents have historically been under-supervised and instructed in the outpatient setting, only a narrow literature documents the perceived importance of bedside teaching. In interviewing 13 internists who taught IM residents in outpatient clinics, Loftus et al. found that over half (54%; 7/13) felt that in-person instruction was important for modeling the physician-patient relationship and for improving history taking and physical examination skills [9]. More germane to our study, Blount et al. asked 1,046 residents to review a list of 38 teaching behaviors and to rank them by importance [10]. “Demonstrating examination skills and findings” was ranked fifth and eighth by PC and non-PC residents, respectively.

Providing more and more consistent clinical oversight and teaching in ambulatory clinics may require substantial investments in the form of increased bedside modeling and time per patient visit, dedicated physical examination didactics, standardized patient exercises, and reconsideration of assessment-driven advancement. However, such efforts may be critically important. Attesting to residents’ examination-focused milestones then advancing them through levels of training without directly observing them does not enhance their clinical skills.

Second, we were struck by our findings that interns were no more likely to be directly observed or offered bedside instruction than were more senior residents, and that they were no less confident in their examination skills than were more seasoned trainees. In Day et al.’s study of 48 encounters between IM residents and standardized patients [11], there were no appreciable differences in interview or physical examination scores between participating interns and PGY2 and PGY3 trainees. Despite this finding, however, one may expect interns to be offered more instruction and oversight than house officers nearing the end of their training, and attending physicians certainly expect residents’ physical examinations to become more accurate and sophisticated as they advance.

One potential explanation for the lack of difference in confidence is that interns “don’t know what they don’t know” while upper-level house officers are more aware of their limitations. However, our findings certainly need to be confirmed in a larger study involving several training programs. While our analyses revealed no significant differences between answers offered by interns and more senior residents, for several reasons, specifically, the small overall n and the single-institution nature of the study, our results may not be generalizable. If they hold in an expanded study, though, graduate IM educational leaders need to consider how and why residents are not gaining confidence in their physical examination skills as they progress through training and why more junior learners are not being offered enhanced oversight and guidance.

Third, we were alarmed by the vast discrepancies between participants’ confidence in examining different organ systems and believe that this study is the first to describe this phenomenon. IM training is largely inpatient, and most admitting diagnoses are cardiovascular, pulmonary, and gastroenterological [12]. Hence, it is understandable that residents are more comfortable performing cardiopulmonary and abdominal examinations than they are performing musculoskeletal, neurologic, and gynecologic examinations. However, diseases of these systems are common in both outpatient and inpatient IM practice, and emphasis must be placed on learning to detect and evaluate them.

Fourth, although the literature suggests that performing examinations through clothing produces artifactual sounds [13], nearly all respondents examined patients while they were fully clothed, and many felt that those examinations were adequate. To our knowledge, only a single study has interrogated the accuracy of physical examinations performed through clothing [13], so this question merits further consideration and study. It would be equally important, however, to determine where and from whom medical trainees learn that examining patients through their clothing is acceptable and sufficient.

Quill found that house officers’ charting and documentation practices were influenced by their attending physicians’ practices [14]. He concluded that ambulatory preceptors’ patient care styles and patterns may shape those of their residents. Future studies should query whether attending physicians’ personal examination practices are adopted by their house officers, and preceptors must ensure that their residents’ patients are being offered appropriate and thorough examinations.

Finally, many respondents felt their attending physicians were uninterested in teaching or lacked the skills to do so. Nearly two-thirds of participants felt that increased engagement by supervising physicians would enhance the quality of their feedback, and 21.1% (12/57) reported that lack of interest by attending physicians limited bedside instruction. Preceptorships are often offered to faculty without assessment of their qualifications or examination skills. Perhaps PDs should grant these positions only to their most skilled and dedicated educators and provide feedback and skill-building to lower-rated attendings.

This preliminary single-institution study has a number of limitations. First, for reasons listed above, it was difficult to fully validate the survey before distribution. Second, the participant pool was relatively small, and not every respondent completed the survey. Third, we were unable, given that the instrument was anonymized, to address potential response bias. Finally, given the small number of responses and the single-institution nature of this initial work, results are not at this point generalizable.

## Conclusions

Our findings, while preliminary, seem to indicate that IM residents are uncomfortable performing several important elements of routine bedside assessments and are inadequately instructed in physical examination skills in the outpatient setting. They also feel that more time allotted per patient visit and increased engagement by supervising physicians would improve their education. These insights alone may encourage IM PDs to conduct assessments such as ours within their own institutions. We hope to expand this work to collaborating residency programs to both confirm our findings and to renew efforts to improve graduate-level clinical instruction.

## Appendices

Physical examination survey for residents 2025

Q1 In which year of your residency are you?

o   PGY1

o   PGY2

o   PGY3

Q2 Are you in the primary care track?

o   Yes

o   No

Q3 What do you think you may do after you complete residency? Please pick only one.

o   Policy/Research

o   Primary care

o   Hospitalist medicine

o   Specialty

o   Training/Fellowship

o   I'm not yet certain

Q4 I identify my gender as:

o   Male

o   Female

o   Transgender

o   Nonbinary

o   Prefer not to disclose

Q5 I tend to examine patients while they’re fully clothed

o   True

o   False

Q6 I examine patients while they’re fully clothed for the following reason(s)

o   I don’t have time to have patients undress or change into a gown

o   Patients tell me they’re uncomfortable undressing or changing into a gown

o   I have been taught that examinations performed on fully clothed patients are adequate and accurate

o   I believe that examinations performed on fully clothed patients are adequate and accurate

o   Other (please specify)

Q7 During clinic sessions, how frequently does an attending physician return to the room with me?

o   Rarely

o   Around half the time

o   Routinely

Q8 During clinic sessions, how frequently does an attending physician watch me perform a physical examination and confirm or correct my findings?

o   Rarely

o   Around half the time

o   Routinely

Q9 During clinic sessions, how frequently does an attending physician provide bedside instruction in physical examination?

o   Rarely

o   Around half the time

o   Routinely

Q10 My physical examination findings are crucial in helping me develop and refine a differential diagnosis:

o   Strongly agree

o   Somewhat agree

o   Neither agree nor disagree

o   Somewhat disagree

o   Strongly disagree

Q11 My physical examination findings are crucial in helping me diagnose my patients’ conditions:

o   Strongly agree

o   Somewhat agree

o   Neither agree nor disagree

o   Somewhat disagree

o   Strongly disagree

Q12 I feel confident in my physical examination skills:

o   Strongly agree

o   Somewhat agree

o   Neither agree nor disagree

o   Somewhat disagree

o   Strongly disagree

Q13 I feel confident in my ability to examine the following organs/systems:

o   HEENT

o   Fundoscopic exam

o   Pulmonary

o   Cardiac

o   Peripheral circulation

o   Abdominal

o   Pelvic/GU

o   Neurological

o   Dermatologic

o   Musculoskeletal

Q14 I feel that I am provided adequate physical examination instruction in my continuity clinic:

o   True

o   False

Q15 I feel that more or enhanced physical examination instruction would help me develop as a physician:

o   True

o   False

Q16 I am reluctant to seek feedback on my physical examination skills, as doing so may reflect poorly on me:

o   True

o   False

Q17 I feel that the following would improve and enhance the quality of feedback I receive about my physical examination skills in continuity clinic (select all that apply):

o   More specific comments and feedback on my New Innovations evaluations

o   More real-time observation with feedback

o   Increased use of standardized patients

o   Increased engagement and involvement by my supervising physicians

o   More time allotted for each clinic visit

o   None. The feedback I receive is sufficient

o   Other (please specify)

Q18 In general, which factors do you think most limit bedside physical examination instruction (select all that apply)?

o   Lack of interest by residents

o   Lack of interest by supervising physicians

o   Lack of time for bedside teaching

o   Lack of knowledge of physical examination skills by supervising physicians

o   None. The instruction is sufficient

o   Other (please specify)

Q19 During continuity clinic, if you feel uncertain about physical examination maneuvers or findings, to whom/which resources would you turn to? (select all that apply)

o   A trusted faculty member who does not precept me in continuity clinic

o   My clinic preceptor

o   A more senior resident

o   An online video or written resource

o   None of the above

o   Other (please specify)

Q20 Would you be interested in receiving more physical examination instruction than you currently receive?

o   Yes

o   No

Q21 Please feel free to add any other comments or to share additional insights

__________________________________________________________________________________________________________________________________________________________________________________________________________________________________________
